# Antibiotic Combination Therapy: A Strategy to Overcome Bacterial Resistance to Aminoglycoside Antibiotics

**DOI:** 10.3389/fphar.2022.839808

**Published:** 2022-02-23

**Authors:** Nuoyan Wang, Jing Luo, Fei Deng, Yasi Huang, Hong Zhou

**Affiliations:** Key Laboratory of Basic Pharmacology, Ministry of Education and Joint Laboratory of International Cooperation, Ministry of Education of Characteristic Ethnic Medicine, School of Pharmacy, Zunyi Medical University, Zunyi, China

**Keywords:** aminoglycoside antibiotics, antibacterial mechanisms, bacterial resistance mechanisms, synergy, side effects

## Abstract

After the first aminoglycoside antibiotic streptomycin being applied in clinical practice in the mid-1940s, aminoglycoside antibiotics (AGAs) are widely used to treat clinical bacterial infections and bacterial resistance to AGAs is increasing. The bacterial resistance to AGAs is owed to aminoglycoside modifying enzyme modification, active efflux pump gene overexpression and 16S rRNA ribosomal subunit methylation, leading to modification of AGAs’ structures and decreased concentration of drugs within bacteria. As AGAs’s side effects and bacterial resistance, the development of AGAs is time-consuming and difficult. Because bacterial resistance may occur in a short time after application in clinical practice, it was found that the antibacterial effect of the combination was not only better than that of AGAs alone but also reduce the dosage of antibiotics, thereby reducing the occurrence of side effects. This article reviews the clinical use of AGAs, the antibacterial mechanisms, the molecular mechanisms of bacterial resistance, and especially focuses a recent development of the combination of AGAs with other drugs to exert a synergistic antibacterial effect to provide a new strategy to overcome bacterial resistance to AGAs.

## 1 Introduction

**FIGURE 1:**
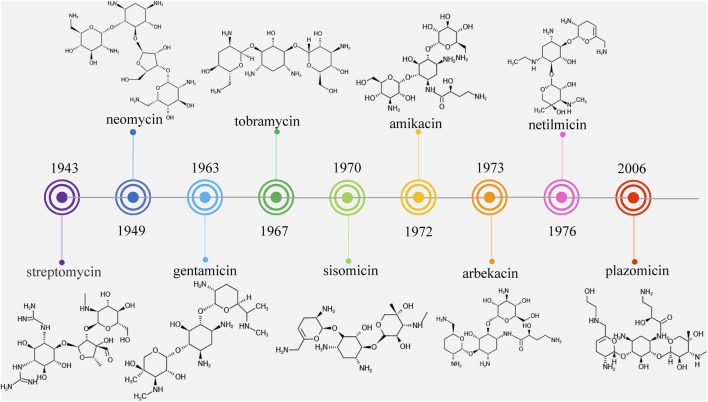
Timeline of aminoglycoside antibiotics development.

**FIGURE 2:**
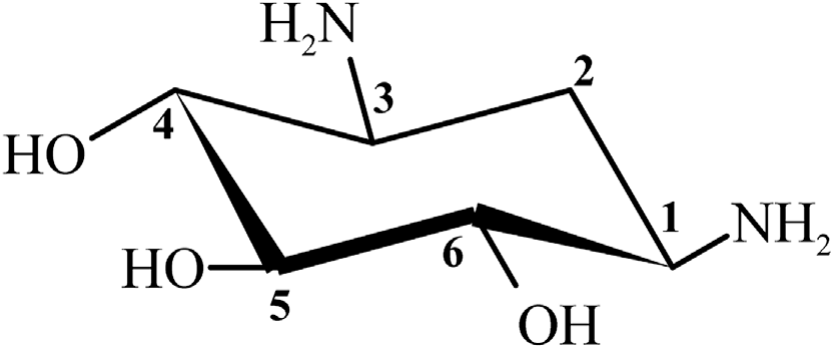
Chemical structure of 2-deoxystreptamine (2-DOS).

Streptomycin was the first discovered aminoglycoside antibiotic (AGA) to be used for tuberculosis treatment in the mid-1940s (Schatz et al., 2005). Thereafter, a series of aminoglycoside antibiotics (AGAs) were discovered, including neomycin (1949), gentamicin (1963), tobramycin (1967), sisomycin (1970), amikacin (1972), and plazomicin (2006) (Figure 1), and all were found to have good antibacterial activities not only for gram-negative bacteria but also for some gram-positive bacteria (Becker and Cooper, 2013; Clark and Burgess, 2020). AGAs are composed of amino sugars and aminocyclic alcohols. 2-deoxystreptamine (2-DOS) is the central unit, which is connected with 2–3 sugar units through glycosidic bonds (Figure 2) (Stead, 2000). According to the structural differences, AGAs are divided into two categories: one is with a 2-deoxystreptamine (2-DOS) core site and another is without a 2-DOS core site (e.g., streptomycin). In turn, according to the substituent linkage position the core site is divided into 4,5-disubstituted 2-DOS and 4,6-disubstituted 2-DOS. The chemical structure of 2-deoxystreptamine is as follows (Figure 2). The structural feature of 4,5 disubstituted 2-DOS AGAs is that the hydroxyl groups at the C-4 and C-5 positions of the 2-DOS ring are substituted and connected to the sugar ring by a glycosidic bond; in 4,6-disubstituted 2-DOS AGAs, the hydroxyl groups at the C-4 and C-6 positions of the 2-DOS ring are substituted and connected to the sugar ring by a glycosidic bond. These sugar units generally have multiple hydroxyl and amino groups, so they have the advantages of good water solubility, good functionality and wide antibacterial spectrum (Johnston et al., 2002).

However, because of the widespread use of these antibiotics, side effects such as nephrotoxicity, ototoxicity and neuromuscular blockade have been found, and bacterial resistance has become more and more frequent. Therefore, the third-generation AGAs such as amikacin (1972), arbekacin (1973), and netilmicin (1976) developed (Kudo and Eguchi, 2016).

AGAs development can be divided into three stages, also known as three generations, based on chemical structure, antibacterial spectrum and resistance to bacterial modification enzymes (Lin, 2001). The first generation of AGAs is represented by kanamycin, which is characterized by the binding of fully hydroxylated amino sugars and cyclic alcohols and no antimicrobial activity against *pseudomonas aeruginosa* (*P. aeruginosa*). The second generation is represented by gentamicin, which contains deoxyamino sugars in the structure and has antibacterial activity against *P. aeruginosa*. The third generation is represented by amikacin, a semi-synthetic derivative of amino cyclic alcohol substituted at the nitrogen position. The third generation of AGAs is characterized by preserving their antibacterial activity while reducing their side effects after modification of their mother nucleus, and has the potential for clinical application due to its characteristics of low drug resistance potential and high plasma concentration (Ristuccia and Cunha, 1982).

However, it was subsequently discovered that these AGAs had limited antibacterial activities against aminoglycoside-modifying enzyme (AME)-producing bacteria. With the development and use of broad-spectrum antibiotics with fewer side effects such as β-lactam antibiotics and fluoroquinolones, the market shares of AGAs decreased and the search for new AGAs has been scaled back. Plazomicin (formerly ACHN-490) is a new type of parenteral drug targeting multidrug-resistant *Enterobacteriaceae*, including AME-producing bacteria, extended-spectrum beta-lactamase (ESBL) and carbapenemase of microorganisms (Haedersdal et al., 2016; Castanheira et al., 2018), which was approved by the Food and Drug Administration (FAD) in June 2018 for the treatment of complicated urinary tract infections (cUTI) and pyelonephritis caused by microorganisms (Saravolatz and Stein, 2020). It became the first FDA-approved AGAs after amikacin was approved in 1981, marking the return of AGAs to the market (Figure 1).

In 2013, the Center for Disease Control (CDC) released the first threat report on antibiotic-resistant (AR) bacteria in the United States. The CDC’s US Antibiotic Resistance Threat 2019 (AR Threat Report 2019) includes up-to-date estimates of the number of deaths and bacterial infections nationwide, re-emphasizing the ongoing threat of antibiotic resistance in the United States. More than 2.8 million infections resulting from antibiotic resistance occur in the United States each year, and more than 35,000 people die from infections. New CDC data show that despite the growing threat of antibiotic resistance in the United States, for example, erythromycin-resistant invasive group A *streptococcus* increased by 351%, drug-resistant *neisseria gonorrhoeae* increased by 124%, and ESBL-producing *enterobacteriaceae* increased by 50%, the number of deaths has decreased since the 2013 report, including an 18% reduction in overall deaths and a 28% reduction in hospital deaths resulting from antibiotic resistance (Antibiotic resistance threats in the United States, 2019, 2019).

According to the CHINET surveillance of bacterial resistance, results of 2020 published in the Chinese Journal of Infection and Chemotherapy in July 2021 (Hu et al., 2021). The susceptibility of clinical isolates from 52 hospitals in major regions of China to antibacterial drugs was tested, and the judgment criteria were based on the 2020 Clinical and Laboratory Standards Institute (CLSI) Drug Sensitivity Judgment Standard (Humphries et al., 2021). In 2020, the proportions of clinical isolates of gram-negative and gram-positive bacteria collected from the above 52 hospitals were 71.9 and 28.1%, respectively.

The resistance rate of gentamicin, a representative drug of AGAs, was calculated on gram-positive coccis and found that, methicillin-resistant *staphylococcus aureus* (MRSA), *enterococcus faecalis* (*E. faecalis*) and *enterococcus* (*E. faecium*) are severely resistant to gentamicin. Most of the methicillin-sensitive staphylococci were sensitive to gentamicin (Table 1) (Hu et al., 2021). After statistics of the bacterial resistance rates to gentamicin and amikacin in *enterobacterales* strains of gram-negative bacilli with high clinical isolation rate, it was found that the resistance of *Klebsiella* to gentamicin and amikacin and *escherichia coli* (*E. coli*) to gentamicin are relatively serious, while *E. coli* is relatively sensitive to amikacin. Among non-fermentative gram-negative bacilli, *P. aeruginosa* was mostly sensitive to gentamicin and amikacin, while *acinetobacter* was severely resistant to the two AGAs (Hu et al., 2021) (Table 2)

**TABLE 1:** Resistance rates of common gram-positive cocci to gentamicin.

Genus names	Bacteria names	Resistance rate (%)
*Staphylococcus* spp.	*Staphylococcus aureus*	20.7
	Methicillin resistant bacteria of *staphylococcus epidermidis*	20.9
	Other *staphylococcus* bacteria (except *pseudointermediate staphylococcus* and *staphylococcus schleiferi*)	25.4
	Methicillin-sensitive strains of *S. aureus*	8.5
	Methicillin-sensitive strains of *S. epidermidis*	5.2
	Other *staphylococcus* bacteria (except *pseudointermediate staphylococcus* and *staphylococcus schleiferi*)	1.3
*Enterococcus* spp.	*E. faecalis*	36.6
	*E. faecium*	45.0

**TABLE 2:** Resistance rates of common gram-negative bacilli to gentamicin and amikacin.

Genus names	Bacteria names	Resistance rate of gentamicin (%)	Resistance rate of amikacin (%)
*Enterobacterale* spp.	*Escherichia coli*	37.4	2.7
	*Klebsiella*	28.9	15.7
Non-fermentative gram-negative bacilli	*Pseudomonas aeruginosa*	8.3	4.5
	*Acinetobacter*	65.3	50.7

## 2 Antibacterial Mechanism of Aminoglycoside Antibiotics

**FIGURE 3:**
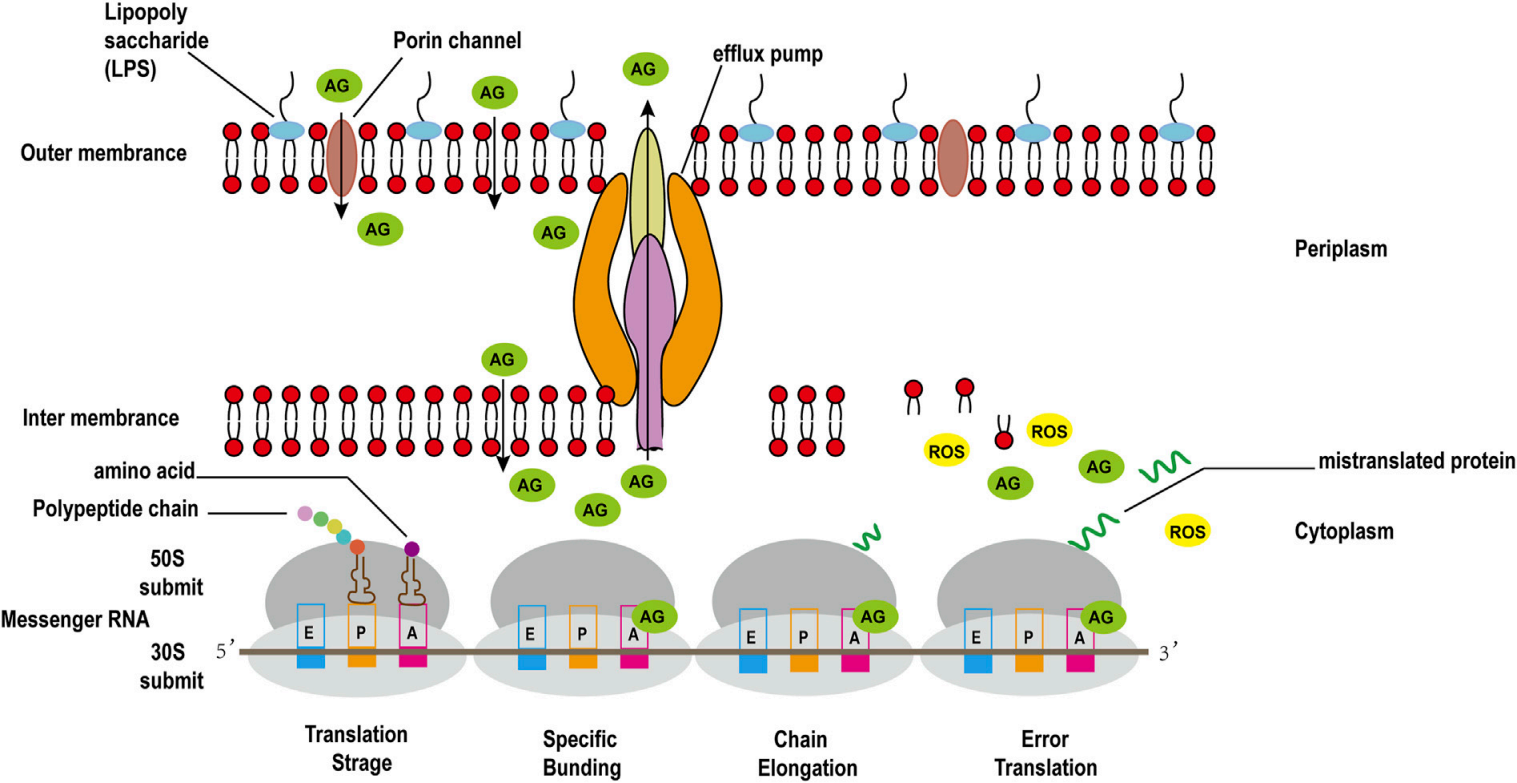
Antimicrobial mechanisms of aminoglycoside antibiotics in gram-negative bacteria.

The binding of 30S ribosomal subunit and tRNA is one of the key step in protein synthesis. Researches have shown that there are three sites where tRNA and 30S subunit bind, comprising Aminoacyl (A), Peptide (P), and Exit (E) sites (Tsai et al., 2013). The potent bactericidal activity of AGAs mainly depends on their specific binding to the A site of the bacterial ribosomal 30S subunit 16S rRNA, interfering with the bacterial intracellular translation process to inhibit bacterial protein synthesis (Degtyareva et al., 2017). However, unlike other commonly used protein synthesis inhibitors, AGAs are bactericidal antibiotics, while common inhibitors of protein synthesis such as chloramphenicol, clindamycin, tetracycline and macrolides are bacteriostatic antibiotics (Serio et al., 2018). Recent studies have discovered that aminoglycoside bactericidal antibiotics act on both gram-negative bacteria and gram-positive bacteria to produce hydroxyl free radicals that are harmful to the bacteria, ultimately leading to their death (Figure 3), while bacteriostatic antibiotics do not promote the production of hydroxyl free radicals (Kohanski et al., 2007).

## 3 Bacterial Resistance Mechanism to Aminoglycoside Antibiotics

### 3.1 Enzymatic Modification of Aminoglycoside Antibiotics

Among various bacterial resistance mechanisms to AGAs, AMEs is the most common mechanism (Wachino et al., 2020). AMEs are divided into three aminoglycoside modifying enzyme families: phosphotransferases (APHs), acetyltransferases (AACs), and adenyl transferases (ANTs) (Sparo et al., 2018). These AMEs use cofactors, acetyl-coenzyme A or ATP to modify NH_3_ or OH groups of AGAs, thereby inactivating them. The genes encoding AMEs are usually carried in plasmids, which promotes the bacterial-bacterial spread of aminoglycoside resistance (Bassenden et al., 2016). Clinically isolated aminoglycoside resistant strains often harbor multiple AME genes, and it is reported that the AME gene is encoded on the same plasmid as the 16S-ribosomal RNA methyltransferases (RMTase) gene (Garneau-Tsodikova and Labby, 2016; López Díaz et al., 2017). The three types of AMEs comprise many subtypes, each with a certain specificity for resistance to AGAs (Garneau-Tsodikova and Labby, 2016).

#### 3.1.1 Acetyltransferases

Acetyltransferases (AACs) acetylate the amino group (-NH_2_) on aminoglycoside molecules, and are members of the N-acetyltransferase (GNAT) superfamily, which are thought to be related to general control non-depressible 5 (GCN5) (Ramirez et al., 2013). The enzyme is divided into four subtypes according to the location of catalytic acetylation: AAC (1), (2′), (3), and (6′) (Ramirez and Tolmasky, 2010).

AAC (6′) enzymes are widespread in gram-negative as well as gram-positive bacteria and are by far the most common, and their genes have been found in plasmids and chromosomes, and are part of mobile genetic elements (Tolmasky, 2000; Centrón and Roy, 2002; Soler Bistué et al., 2008). A group of gram-negative and gram-positive bacteria caused by nosocomial infections called as ESKAPE (*E. faecium*, *staphylococcus aureus* (*S. aureus*), *klebsiella pneumoniae* (*K. pneumoniae*), *acinetobacter baumannii*, *P. aeruginosa*, and *enterobacter*) are highly resistant to antibiotics including AGAs (Rice, 2008). The most common gram-negative member of the AME genes they carry is *aac (6′) -Ib*(Ramirez and Tolmasky, 2010; Shaul et al., 2011; Herzog et al., 2012). N-acetyltransferase AAC (3)-I was established as a selectable marker for gentamicin-based oomycete transformation, and it was found that N-acetyltransferase AAC (3)-I was responsible for gentamicin resistance in *phytophthora palmivora* and *phytophthora infestans* (Evangelisti et al., 2019).

#### 3.1.2 Phosphotransferases

Phosphotransferase (APH) enzymes transfer a phosphate group from ATP to the hydroxyl group (-OH) on the aminoglycoside molecule, and comprise seven subtypes: APH (2″), (3′), (3″), (4), (6), (7″), and (9). The *aph (6) -Id* gene was first discovered in plasmid RSF1010, an 8,684 bp wide host range multicopy plasmid RSF1010 that can replicate in most gram-negative and gram-positive actinomycetes (Meyer, 2009), and this plasmid is also the first source of another APH- *aph (3″) -Ib*, adjacent to *aph (6) -Id* (Daly et al., 2005; Gordon et al., 2008). Due to the spread of this DNA fragment, *aph (6) -Id* and aph *(3″) -Ib* genes were found in both gram-positive and gram-negative bacteria (Ramirez and Tolmasky, 2010). APH (3′) enzymes induce resistance to amikacin, kanamycin and neomycin of AGAs (Aggen et al., 2010; Ramirez and Tolmasky, 2010; Miró et al., 2013).

#### 3.1.3 Adenyl Transferases

Adenyl transferase (ANT) enzymes transfer the adenosine monophosphate (AMP) group in ATP to the hydroxyl group (-OH) of aminoglycoside molecules. There are five classes of this of enzyme: ANT (2″), (3″), (4′), (6), and (9). ANT (2″)-Ia is an enzyme normally encoded by plasmids and transposons, widely distributed as gene cassettes in class 1 and class 2 integrons (Vakulenko and Mobashery, 2003; Ramírez et al., 2005). This enzyme mediates resistance to gentamicin, tobramycin, dibecacin, sisomicin and kanamycin of *Enterobacteriaceae* and non-fermentative gram-negative bacilli (Ramirez and Tolmasky, 2010). Compared with AACs and APHs, ANTs are found less frequently in *enterobacteriaceae* and *P. aeruginosa*, but still act as an important role of bacterial resistance to AGAs (Almaghrabi et al., 2014; Holbrook and Garneau-Tsodikova, 2018).

### 3.2 Decreased Drug Accumulation

#### 3.2.1 Increased Efflux Pumps

The active efflux of antibiotics from bacteria plays an important role in various antibiotic resistance mechanisms. It can also interact with other drug resistance mechanisms such as membrane permeability changes, enzymatic modification of antibiotics, and changes to the target of antibiotic action, thereby significantly increasing antibiotic resistance levels (Li et al., 2015). In *P. aeruginosa*, the MexXY-OprM system confers low-level intrinsic resistance to AGAs, and mutations to the mexZ repressor, which are expressed from the *mexXY* operon, induce widespread AGAs resistance in clinical isolates (Poole, 2011). In *E. coli* and other members of *Enterobacteriaceae* spp., the AcrAD-TolC system cause the efflux of AGAs and other antibiotics (Li et al., 2015; Garneau-Tsodikova and Labby, 2016). Perhaps because of the significance of AMEs in the mechanism of AGAs resistance, the clinical resistance of *Enterobacteriaceae* spp. to AGAs through active efflux pumps is rarely reported.

#### 3.2.2 Reduced Bacterial Outer Membrane Permeability

##### 3.2.2.1 Mutations of Genes Encoding Porins

To take up nutrients, bacteria have evolved many absorption mechanisms. One of which is the formation of water-filled pores extending through the membrane to promote the absorption of hydrophilic compounds. The proteins that form these channels are called porins. Drug resistance caused by porins mutations include: decreased expression, no expression, and structural mutations. Antibiotic resistance caused by changes in porin expression and their mutations might be a limited resistance mechanism because they can also lead to reduced nutrient intake by bacteria (Garneau-Tsodikova and Labby, 2016). AGAs have a hydrophilic structure and a positive charge; therefore, they are believed to penetrate bacterial cell walls through porin channels instead of directly diffusing through the phospholipid bilayer (Nikaido and Pagès, 2012). Beta-lactams, fluoroquinolones, tetracycline antibiotics, and other antibiotics also enter the bacterial membrane through porins, and the loss of porins on the membrane will lead to resistance to these antibiotics. It was found that mutants of *E. coli* lacking porin OmpF are resistant to AGAs (Bafna et al., 2020). However, the survival rate of the OmpF mutant of *E. coli* is lower than that of the wild-type; thus it might not be widespread in clinically isolated strains (Zhao et al., 2014).

##### 3.2.2.2 Membrane Protease and Other Functional Proteins

Membrane protease, as a member of the protein synthesis system, is used to identify and degrade misfolded and translated proteins. As AGAs accumulate in bacteria, they will produce mistranslated proteins. Membrane proteases recognize and remove them, thereby producing resistance to AGAs. Studies have shown that proteolysis plays a central role in the mechanism of the intrinsic resistance to AGAs of *P. aeruginosa*, in which the membrane protease FtsH is particularly important (Hinz et al., 2011). Deletions of genes related to lipid biosynthesis or metabolism (lptA, faoA), phosphate uptake (pstB), two-component regulators (amgRS, PA2797-PA2798), and a gene with unknown function (PA0392) also increased the sensitivity to AGAs, such as gentamicin, confirming their contribution to intrinsic resistance (Krahn et al., 2012).

#### 3.2.3 Biofilm Formation

The ability of bacteria to form biofilm enhances their ability to infect the host, which is usually associated with chronic infections by invasive bacteria (Yarlagadda and Wright, 2019). A biofilm is a collection of microorganisms attached to the surface of living organisms or non-living organisms, and is embedded in the matrix of extracellular polymeric substances (EPSs), including proteins, extracellular polysaccharides, and extracellular DNA (eDNA) (Das et al., 2013). The eDNA produced during cell lysis is an important part of the biofilm. The eDNA in *P. aeruginosa* can acidify the environment and induce the function of the PhoPQ and PmrAB two-component regulatory system to regulate gene expression, thereby enhancing aminoglycoside resistance (Wilton et al., 2016). The complex structure of a biofilm can protect the bacteria from the host’s immune system and antibacterial drugs (Lebeaux et al., 2014). The general mechanisms by which biofilm-mediated resistance protects bacteria from antibiotic attack include preventing antibiotic penetration, altering the microenvironment to induce slow growth of biofilm cells, induction of adaptive stress responses, and sustained cell differentiation (Stewart, 2002). Biofilm-mediated drug resistance is an adaptive drug resistance, when bacteria lose biofilm protection, antibiotic susceptibility can be rapid (Walters et al., 2003). Chronic infections caused by *P. aeruginosa* have been found to be usually accompanied by biofilm formation (Hall and Mah, 2017). While the adaptive resistance mechanism of *P. aeruginosa* infection and recurrence is related to biofilm-mediated resistance (Pang et al., 2019).

### 3.3 Modification of Drug Targets

#### 3.3.1 Methylation of 16S rRNA Ribosomal Subunit

One mechanism of clinically significant drug resistance is acquired 16S rRNA methyltransferase, which adds methyl groups to specific residues in the 16S rDNA site using S-adenosylmethionine (SAM) as a cofactor. Compared with the original 16S rRNA, the affinity of AGAs for 16S rRNA added with methyl groups is reduced, which confers high-level and broad-spectrum aminoglycoside resistance (Wachino et al., 2020).

16S-RMTases are divided into two categories: Those with the N7 position of methylated nucleotide G1405 and those with N1 position of methylated nucleotide A1408. Subsequently, a total of nine N7-G1405 16S-RMTases) were identified, which include ArmA, RmtA, RmtB (including RmtB1 and RmtB2 alleles), RmtC, RmtD (including RmtD1 and RmtD2 alleles), RmtE, RmtF, RmtG, and RmtH. These N7-G1405 16S-RMTases have moderate to high amino acid sequence similarity (Doi et al., 2016). However, N7-G1405 16S-RMTases are resistant to 4,6-disubstituted 2-DOS, but not to 4,5-disubstituted 2-DOS and other AGAs, with ArmA, RmtB, and RmtC 16S-RMTases having a high level of resistance to 4,6-disubstituted 2-DOS AGAs. There is only 30% amino acid identity among the three enzymes; however, they show a high degree of three-dimensional structural similarity (Nosrati et al., 2019).

#### 3.3.2 rRNA Sequence Mutations

Aminoglycoside resistance is usually not caused by bacterial target mutations, because most bacteria have more than one copy of the rRNA coding gene. To induce aminoglycoside resistance through this mechanism requires mutations in every gene copy (Serio et al., 2018). AGAs affect ribosome mutation to produce drug resistance via the *rrs* and *tlyA* genes. The *rrs* gene, encoding 16S rRNA (Honoré et al., 1995), and the *tlyA* gene, encoding a 2′-O-methyltransferase, an modifies nucleotide C1409 in Helix 44 of 16S rRNA and nucleotide C1920 in Helix 69 of 23S rRNA, respectively (Johansen et al., 2006). It was found that the most common mutations related to AGA resistance in clinically resistant *mycobacterium tuberculosis* were A1401G, C1402T, and G1484T in the *rrs* gene (Georghiou et al., 2012).

#### 3.3.3 Ribosomal Mutations

The bacterial ribosome is composed of a small 30S subunit composed of 16S rRNA and 20 proteins, and a large 50S subunit composed of 23S rRNA, 5S rRNA and 34 proteins. AGAs bind to a region of the 16S rRNA decoding region on the ribosome 30S subunit, then disrupting the normal assembly of the protein, thereby finally affecting bacterial reproduction (Wilson, 2014).

Some 2-DOS AGAs such as gentamicin, neomycin B and paromomycin bind to the major groove of helix 69 (H69) of the 23S rRNA of the 50S large subunit, thereby affecting the translation on ribosomes (Wang et al., 2012; Wasserman et al., 2015). In AGA-resistant *P. aeruginosa*, changes in the ribosomal helix 69 conformation and deletion of the ribosomal protein UL6 will prevent them from binding to the translation initiation factor IF2, thus interfering with protein synthesis (Halfon et al., 2019).

### 3.4 Changes of Bacterial Metabolism

The carbon source and cellular respiration play important roles in bacterial pathogenicity and antibiotic sensitivity (Vitko et al., 2015; Vilchèze et al., 2017). Crc, Hfq and a small RNA CrcZ act as central regulators in carbon metabolism of *P. aeruginosa*. The catabolite repression control protein (Crc) forms a complex with the RNA chaperone Hfq, and directly inhibits the translation of catabolic genes via binding to the target mRNA (Sonnleitner et al., 2018). Hfq is considered critical for biofilm formation (Pusic et al., 2016), and also contributes to bacterial resistance to antibiotics such as gentamicin (Pusic et al., 2018).

The triphosphate isomerase, TpiA, can reversibly convert glyceraldehyde 3-phosphate to dihydroxyacetone phosphate, which is a key step linking glucose metabolism to glycerol and phospholipid metabolism. Mutations in the *TpiA* gene have been found to enhance carbon metabolism, respiration, and oxidative phosphorylation in bacteria, thereby increasing the membrane potential and promoting AGA uptake. Studies have found that the level and stability of CrcZ in bacteria with *TpiA* mutations are increased. *CrcZ* mutations can restore the expression of type III secretion system (T3SS) genes and enhance the resistance of bacteria to AGAs (Xia et al., 2020).

## 4. Strategies to Reverse Bacterial Resistance to Aminoglycoside Antibiotics

### 4.1 Structure Optimization and Modification Strategy of Aminoglycoside Antibiotics

To solve the problem of resistance to AGAs, except the development of new antibiotics, structural modification of existing AGAs to restore their antibacterial activity remains an important way. In the case of Aminoglycoside modified enzymes, AMEs modification is one of the important mechanisms conferring resistance to AGAs in bacteria (Serpersu et al., 2008). There are genes encoding AMEs in the plasmid (Bassenden et al., 2016), while AMEs genes catalyze aminoglycoside hydroxyl or amine functionalities modification, rendering AGAs cannot bind to bacterial ribosomal targets, resulting in drug resistance (Ramirez and Tolmasky, 2010). For example, second-generation semi-synthetic AGAs derivatives have been designed with reference to the ribosome structure and the mechanism of AMEs, including dibekacin and amikacin modified from kanamycin; isepamicin and netilmicin modified from gentamicin; and sisomicin. At present, research is focusing on the third-generation AGAs that block the site of action of AMEs and have reduced side effects. Plazomicin (ACHN-490) is a modified sisomicin derivative; it was developed as an agent to avoid resistance via common AMEs (Dozzo and Moser, 2010). The general structure of AGAs is characterized by the presence of aminocyclic alcohol nuclei (cyclohexanes with or without substituted amino and hydroxyl groups), which are usually coupled to AGAs (Ramirez and Tolmasky, 2017). Plazomicin blocks the AME site of action through a series of structural modifications to enhance its antibacterial activity. As plazomicin lacks hydroxyl groups at the 3 and 4′ positions, it protects itself from modification of ANT (4′) and APH (3). The introduction of an unsaturated hydroxyethyl group at position 6′-NH3 can block the action site of AAC (6′). Plazomicin also has an N-1 substitution with 4-amino-2-hydroxybutanoic acid, which blocks AAC (3), ANT (2″), and APH (2″). These AMEs are often present in ESBL-producing and carbapenem-resistant *Enterobacteriaceae*, playing an important role in resistance to AGAs (Karpiuk and Tyski, 2015; Syue et al., 2016; Eljaaly et al., 2019).

### 4.2 Synergistic Antibacterial Strategy of AGAs Combined With Other Drugs

In addition to bacterial resistance, side effects of AGAs are another reason that hinders the clinical application of AGAs. The represent toxic side effects include ototoxicity caused by AGAs, cochlear nerve injury caused by ototoxicity and nephrotoxicity caused by proximal tubular epithelial cell injury (Kros and Steyger, 2019; Jospe-Kaufman et al., 2020; Rosenberg et al., 2020). However, the antibacterial effect of AGAs in combination with different compounds is better than that when the antibiotics are used alone. The investigation found that the combination cannot only exert a synergistic antibacterial effect but also reduce the dosage of the AGAs, leading to the reduced chance of occurrence and degree of side effects. Herein, synergistic antibacterial effects in combination with AGAs reported in recent years are summarized below.

#### 4.2.1 Combination With Compounds With Antibacterial Activities

##### 4.2.1.1 Combination With Other Antibacterial Agents

###### 4.2.1.1.1 Beta-Lactam Antibiotics

Combinations of AGAs such as gentamicin can expand the scope of clinical treatment, accelerate bacterial clearance, and improve antibiotic resistance, especially the because of the synergistic antibacterial effects with β-lactam antibiotics (Le et al., 2011; Rhodes et al., 2017). β-lactam antibiotics cause non-fatal damage to the bacterial cell wall, thus promoting AGA entry inro bacteria and enhancing their killing ability (Davis, 1982). AGAs in combination with β-lactam antibiotics are commonly used in severe hospital-acquired infections by multidrug resistant species, such as acquired pneumonia, ventilator-associated pneumonia, and sepsis (Tamma et al., 2012; Sick et al., 2014; May 2016).

###### 4.2.1.1.2 Macrolides

Azithromycin is the most commonly used macrolide in clinical practice, which has the advantages of strong antibacterial activity and a long half-life. AGAs and azithromycin are used widely to treat *P. aeruginosa* infections, and the combination of the two antibiotics can reduce the single-dose of each antibiotic. Studies have found that gentamicin can inhibit translation and increase the killing effect of azithromycin on planktonic and biofilm cells (Ren et al., 2019). Previous studies have shown that gentamicin or gemfloxacin, combined with azithromycin, has a significant effect on the treatment of genitourinary gonorrhea (Kirkcaldy et al., 2014).

###### 4.2.1.1.3 Polymyxins

Polymyxins are one of the most commonly used drugs against gram-negative bacteria, especially against *P. aeruginosa*. The combination of a polymyxin and amikacin presented a synergistic antibacterial effect on polymyxin-sensitive and resistant *P. aeruginosa*. Polymyxin-sensitive *P. aeruginosa* (FADDI-PA111) and polymyxin-resistant *P. aeruginosa* (LESB58) were selected for the combined antibacterial treatment with polymyxin B and amikacin. After their co-administration, a metabonomic analysis showed that the necessary bacterial membrane lipid levels involved in the biosynthesis of phospholipids and lipopolysaccharide (LPS) were reduced significantly, demonstrating that polymyxin B combined with amikacin could inhibit the intermediates in LPS synthesis significantly (Hussein et al., 2019). The pentose phosphate pathway (PPP) is the key source for the synthesis of LPS precursors, and the antibacterial effect might result from early inhibition of the PPP. The study also found that polymyxin B combined with amikacin interfered with the tricarboxylic acid (TCA) and glycolysis pathways in FADDI-PA111, but this effect was not found in LESB58. Bacterial central carbohydrate metabolism, represented by glycolysis and TCA cycle, has been an important target in research into new antibiotics in recent years. The inhibitory effect of polymyxin B combined with amikacin on the pyridine nucleotide cycle (PNC) might also be one of the mechanisms of its synergistic antibacterial effect. The combined treatment resulted in a significant reduction of D-ribose-5-phosphate, a key initial intermediate for purine and pyrimidine metabolism in FADDI-PA111, indicative of nucleotide degradation. The combination of the two drugs had a significant effect on the metabolism of arginine and proline in FADDI-PA111, leading to the destruction of amino acid pathways (Hussein et al., 2019). The disruption of amino acid pathways, represented by arginine metabolism, is considered to be a new way to deal with bacterial infection and destroy their mechanism of action (Xiong et al., 2016).

###### 4.2.1.1.4 Antimicrobial Peptides

AMPs have emerged from their origins as antibiotics in nature and are widely distributed in animals and plants. They are the first line of defense against pathogens invading the body (Yeung et al., 2011). AMPs have the characteristics of broad-spectrum resistance, rapid action, and low resistance tendency. With the increasing resistance to existing antibiotics, AMPs are expected to become a more widely used class of antibacterial drugs (Wang et al., 2014). In the study of the synergistic antibacterial effect of antimicrobial peptide PMAP-36 or PRW4 combined with gentamicin against *E. coli* and *S. aureus*, it was found that AMPs and gentamicin had a synergistic (fractional lethal concentration (FLC) < 0.5) and a partial synergistic effect (0.5 < FLC < 1) against *E. coli* and *S. aureus*, respectively. The synergistic antibacterial effect of gentamicin with PMAP-36 or PRW4 on gram-negative bacteria (*E. coli*) was found to be caused not only by the AMPs weakening the bacterial outer membrane and increase membrane permeability, so that gentamicin could more easily enter the cytoplasmic membrane, but also because of their direct antibacterial activity via interactions with intercellular targets, such as DNA, after entering the bacteria. The exact mechanism of AMPs in gram-positive bacteria is still unclear. Analyzing the molecular structure of the two AMPs, it was noted PMAP-36 is more hydrophobic than PRW4, and this hydrophobicity might promote membrane rupture and increase the antibacterial effect (Wang et al., 2014).

##### 4.2.1.2 Combination With Other Natural Plant Extracts With Antibacterial Activities

Plant essential oil has antibacterial, synergistic antibacterial and other pharmacological effects; they are one of the important sources of natural medicine extracts (Bakkali et al., 2008). Therefore, during the development of higher antibacterial activity against a variety of gram-negative bacteria and gram-positive bacterial infections with antibacterial drugs combined with plant essential oils, it was found that gentamicin combined with the essential oils extracted from *Pelargonium graveolens* and *Aniba rosaeodora* had synergistic effects. The combination can reduce the minimum effective dose of gentamicin. Especially in the essential oil of *Acinetobacter baumannii*, gentamicin combined with this had a significant synergistic antibacterial effect. The chemical composition of essential oils was studied using gas chromatographic analysis, which showed that the essential oils extracted from P*elargonium graveolens* and *Aniba rosaeodora* contained a high percentage of terpene alcohols (Rosato et al., 2010). The antibacterial mechanism of monoterpenes was found to be due to the destruction of the microbial plasma membrane, resulting in changes in membrane permeability and intracellular substance efflux (Trombetta et al., 2005), such that a high content of terpene alcohols benefitted the antibacterial effect of gentamicin, mainly due to the blocking protein synthesis by binding to the 30S subunit of the bacterial ribosome.

*Camellia sinensis* (green tea) has been proved to have a wide range of antibacterial activity. A study found a high content catechin especially epigallocatechingallate (EGCG) in green tea (Song and Seong, 2007). Studies have found that EGCG combined with gentamicin has a strong synergistic antibacterial effect against multi-drug resistant *E.coli* and *S. aureus*, because EGCG in green tea can destroy the bacterial cell membrane and eventually lead to bacterial rupture (Parvez et al., 2019).

Triphala, comprising the dried pericarp of the fruit of three plant from India, *Myrobalans Terminalia chebula Retz*. (Haritaki, Family: Combretaceae), *Terminalia bellirica Roxb.* (Bibhitaki, Family: Combretaceae), and *Phyllanthus emblica Linn.* or *Emblica officinalis Gaertn.* (Amalaki or the Indian gooseberry, Family: Euphorbiaceae), is an important medicine in the Indian traditional medicine system (Baliga, 2010; Baliga et al., 2012). Triphala and its single drug extracts have certain antibacterial activity against gram-negative and gram-positive bacteria (Srikumar et al., 2007; Tambekar and Dahikar, 2011; Parveen et al., 2018). Its phenolic content is closely related to its antibacterial activity (Bag et al., 2013). A study showed that gentamicin and triphala have a synergistic antibacterial effect on some multidrug resistant gram-negative bacilli. Phenolic compounds can destroy the cell membrane of the bacteria, thus, in the synergistic antibacterial effect of gentamicin and triphala. The mechanism may be similar to the synergistic effect of gentamicin and β-lactam antibiotics (Manoraj et al., 2019).

Brazilian red propolis contains a large number of phenolic compounds, of which the largest proportion is flavonoids, which are believed to be related to the antibacterial effect of red propolis (F. B. Carvalho et al., 2016; da Cruz Almeida et al., 2017). Previous studies have found that combinations of antibiotics with phenolic compounds cause damage to the bacterial cell wall or cell membrane and enhance antibiotic activity (Mun et al., 2013; Guo et al., 2015), making it easier for antibiotics to enter the interior of the bacterial cell. This might be the main mechanism of action by which propolis compounds enhance antibiotic activity (Orsi et al., 2012). Studies have reported the synergistic effect of gentamicin or imipenem combined with Brazilian red propolis on *S. aureus* and *P. aeruginosa*, and discovered the effect of seasonal humidity changes on resin plants: The levels of active ingredients in propolis in specimens collected in dry season period were obviously higher than those in specimens collected in the rainy season. Therefore, the study found that the combination of propolis collected in the drier period and gentamicin or imipenem has a synergistic effect (Regueira et al., 2017).

5-hydroxy-3,7,4-trimethoxyflavone (VG.EF.CLII) was isolated from *Vitex gardneriana* leaves. Nuclear magnetic resonance analysis found that the isolated 5-hydroxy-3,7,4 - trimethoxyflavone had the chemical formula C_18_H_16_O_6_. To study its antibacterial activity against *E. coli* and *S. aureus*, the minimum inhibitory concentration (MIC) was used to evaluate the antibacterial effect of the component and its ability to regulate the activity of antibiotics. VG.EF.CLII showed antibacterial activity against multidrug resistant bacterial strains. When combined with the antibiotics norfloxacin and gentamicin, respectively, and tested against 27 strains of *E. coli* and 358 strains of *S. aureus* isolated from surgical wounds, VG.EF.CLII had a significant synergistic antibacterial effect (Macedo et al., 2019).

#### 4.2.2 Combination With Other Compounds or Materials Without Antibacterial Activity

##### 4.2.2.1 Combination With Anti-Inflammatory Analgesics

Periprosthetic joint infection (PJI) is a serious complication of total joint replacement. Antibacterial drugs, generally gentamicin, tobramycin and vancomycin, are chosen for local and systemic administration against PJI (Hickson et al., 2015; Vrabec et al., 2016). Some analgesics have been found to also possess certain antibacterial activity against a variety of pathogens (Johnson et al., 2008). Several types of analgesics, such as sodium channel blockers, non-steroidal anti-inflammatory drugs (NSAIDs) and opioids, are used in pain management after arthroplasty (Imani, 2011). The synergistic antibacterial effects of bupivacaine, lidocaine (sodium channel blocker) and ketorolac (NSAID) in combination with gentamicin (broad-spectrum antibiotic) against *S. aureus* were assessed. The results showed that the fractional inhibitory concentration index (FICI) of the combination of ketorolac and gentamicin was lower than 0.4, indicating that the combination had a synergistic antibacterial effect (Gil et al., 2020).

In multidrug-resistant bacteria, other non-antibiotic drugs can be used in combination with drug-resistant antibiotics to regain the antibacterial effect (Cai et al., 2007; Chan et al., 2017). In addition to the common anti-inflammatory, analgesic, and antipyretic effects of NSAIDs, there are also reports showing that they have a synergistic effect on the antibacterial activity of antibiotics (Yin et al., 2014; Obad et al., 2015; Chan et al., 2017). A study found that in the treatment of *MRSA* infections using NSAIDs combined with drug-resistant antibiotics, it was found that Meloxicam had a partial synergistic effect with oxytetracycline and gentamicin. In addition, Flunixin Meglumine had a synergistic effect with oxytetracycline and a partial synergistic effect with gentamicin; however, the mechanism of action needs further study (Altaf et al., 2019).

##### 4.2.2.2 Combination With and Natural Plant Extracts Without Antibacterial Activities

As mentioned above, plant essential oils have antibacterial, synergistic antibacterial and other pharmacological effects. Although the essential oil of *Mikania cordifolia* (EOMc) and its major constituent, limonene, has no direct antibacterial activity, it can reverse antibiotic resistance by regulating the action of antibiotics. For *P. aeruginosa*, EOMc combined with gentamicin and norfloxacin displayed a synergistic antibacterial effect; and for *S. aureus*, EOMc reduced the MIC of gentamicin toward bacteria (Justino de Araújo et al., 2020).

Considering that saponins can alter the local chemical environment of the cell membrane and may alter how bacteria absorb or act on antibiotics, the combination of glycyrrhizic acid and gentamicin was found to have therapeutic potential against local bacterial infections caused by vancomycin-resistant *enterococci* (Schmidt et al., 2016). The Chinese herbal compound, plumbagin, stimulates the uptake of gentamicin by carbapenem-resistant *klebsiella pneumonia* (CRKp) by enhancing the efflux of the TCA cycle and the proton-motive force to achieve synergistic antibacterial activity. It can be combined with aminoglycoside antibiotics to enhance its efficacy and reduce its dosage (Chen et al., 2020).

*Croton ceanothifolius* essential oil (CcEO) can enhance the antibacterial activity of gentamicin and norfloxacin against *E. coli*, *S. aureus*, and *P. aeruginosa*. The presence of the two antibiotics acting within the bacterial cell indicated that the CcEO promotes the penetration of antibiotics into the bacterial cytoplasm and thus exerts a synergistic effect. By measuring the MIC of CcEO against multidrug resistant bacteria, it was found that the MIC was greater than 1,024 μg/ml in all strains, indicating that CcEO has no antibacterial activity. CcEO was analyzed by gas chromatography-mass spectrometry (GC/MS), and a total of 25 chemical components were identified. The main ingredients include bicyclogermacrene (26.3%), germanene D (14.7%), and E-caryophyllene (11.7%). The authors hypothesized that although CcEO cannot exert a direct antibacterial effect, it can synergistically enhance the effect of antibiotics on membrane permeability (Araújo et al., 2020).

Kaempferol 7-O-β-D-(6″-O -cumaroyl)-glucopyranoside flavonoids were isolated from croton leaves and studied for their antibacterial and synergistic antibacterial effects. It was found that although it did not have direct antibacterial activity against *S. aureus*, *E. coli*, and *P. aeruginosa*, when kaempferol 7-O-β-D-(6″-O-cumaroyl)-glucopyranoside at 128 μg/ml was combined with gentamicin, it showed a synergistic antibacterial effect on both *S. aureus* and *E. coli* (Cruz et al., 2020). The hydroxyphenyl groups in flavonoids have affinity for proteins; therefore, these compounds act as inhibitors of bacterial enzymes and interfere with their synthesis pathways (Alcaráz et al., 2000).

The Hexanic Zea Mays L. Silk Extract (Poaceae or HEZN) is used widely in Brazilian folk medicine to treat genitourinary tract diseases. A study used standard *E. coli* ATCC 25922, *S. aureus* ATCC 25923 and *P. aeruginosa* ATCC 27853 strains, as well as 27 multi-resistant strains of *E. coli*, 35 strains of *S. aureus,* and 31 strains of *P. aeruginosa*. The authors found that HEZM and its metabolites had an MIC ≥1,024 μg/ml for all tested strains, indicating that at least for these strains, HEZM and its metabolites have no obvious antibacterial activity. In order to study the regulatory effect of HEZM on antibiotics, a subinhibitory concentration (MIC/8) combined with amikacin and gentamicin was selected to test the resistance of multiple drug-resistant strains. In *S. aureus* and *P. aeruginosa*, HEZM combined with amikacin and gentamicin reduced the MICs of the antibiotics significantly. However, the binding of the extract with amikacin against *E. coli* significantly increased the MIC of this antibiotic, indicating that HEZM antagonized the effect of the antibiotic (A. B. L. Carvalho et al., 2019). Phytochemical analysis of HEZM revealed that it contained four types of important secondary metabolites, including: tannins, flavonoids, flavonols, and xanthones (A. B. L. Carvalho et al., 2019). Flavonoids, as common extracts in natural plants, are most frequently related to the synergistic effects of natural products. One of the classes of metabolites in the extract is xanthones, and a subgroup of xanthones, called oxygenated xanthones, can increase the solubility of cell membranes to exert antibacterial activity in norfloxacin-resistant *S. aureus (* *Xiao et al., 2008* *)*. Studies have found that the effects of HEZM on AGAs are different in different gram-negative and gram-positive bacteria. The structural differences between gram-negative and gram-positive bacteria might also determine the effect of natural extracts on antibiotics (A. B. L. Carvalho et al., 2019).

##### 4.2.2.3 Combination With Nanomaterials

Nanomaterials especially silver nanoparticles (AgNPs) have a synergistic antibacterial effect when combined with antibiotics (Hajipour et al., 2012; Singh et al., 2015). Reactive oxygen species (ROS) production is a common mechanism by which antibiotics cause bacterial cell death (Kohanski et al., 2007; Kim et al., 2011). Studies have found that gentamicin significantly enhanced the generation of ROS by AgNPs. Luminol chemiluminescence (CL) proved that the antibacterial activity of Tween-stabilized AgNPs is accompanied by the production of ROS. Gentamicin combined with Tween-stabilized AgNPs showed synergistic antibacterial activity against gentamicin-resistant *staphylococcus epidermidis* (Mazur et al., 2020). AgNPs combined with antibiotics can kill bacteria via different mechanisms, thereby playing a synergistic antibacterial effect (Sukhorukova et al., 2018). It has been reported that AgNPs have the ability to penetrate the bacterial cell wall, destroy the cell membrane, and cause the death of the bacteria. In *E. faecalis (Ef)* infection, we also tested the synergistic antibacterial effect of multiple antibiotics and AgNPs, and found that the synergistic antibacterial effect of gentamicin and chloramphenicol combined with AgNPs was significant (Katva et al., 2018).

Graphene is a biocompatible material with biocarrier, cancer therapy, biosensing and antibacterial activities (Zhou et al., 2009; Yang et al., 2010; Zhang et al., 2010; Liu et al., 2011). In the study of the antibacterial effect of water-soluble TOB-GO-Ag composite composed of aminoglycoside antibiotics (tobramycin), Graphene oxide (GO) and AgNPs against multidrug-resistant gram-negative *E. coli*, it was found that compared with GO, AgNPs and tobramycin, TOB-GO-Ag showed the highest antibacterial activity. The research of the mechanism by which TOB-GO-Ag composite exerted a synergistic antibacterial effect via destroying the integrity of the cell wall and promoting the entry of Ag^+^ ion and GO into cells, which finally destroyed bacteria through oxidative stress mechanism. Meanwhile tobramycin in the prepared composites could also help affect bacterial growth by inhibiting protein synthesis (Ullah et al., 2018).

Some bismuth (Bi)-containing compounds have been reported to have synergistic antibacterial effects toward bacteria when used in combination with antibiotics (Keogan and Griffith, 2014). The study found that the MIC values of Bi_2_S_3_ nanoparticles against *S. aureus* and MRSA are all >1,024 μg/ml, indicating that Bi_2_S_3_ has no antibacterial activity against these two bacteria, but combined with gentamicin has a synergistic antibacterial effect in MRSA. The synergistic antibacterial mechanism was mainly related to the interaction between Bi_2_S_3_ and gentamicin, the destruction of bacterial cell membrane, the increase of gentamicin content in bacteria and the generation of ROS in bacteria (Ma et al., 2017).

CaCO_3_ nanoparticles (CCNPs) have a relatively regular chain structure, and the crystal grain size is about 62.5 nm. Studies have found that CCNPs can carry gentamicin and prolong the release time of gentamicin; so that the complete release time of gentamicin can be extended to 24 h. CCNPs can improve the antibacterial activity of gentamicin significantly. Zeta potential analysis and microscopic observations showed that CCNPs adsorbed on the surface of bacteria increased the degree of damage to the bacterial cell wall, enhanced the permeability of the cell membrane, leading to the increased bacterial death (Pan et al., 2017).

##### 4.2.2.4 Combination With Photosensitizers

Some antibiotics combined with photosensitizer-mediated photodynamic antimicrobial chemotherapy (PACT) have been found to be with better antibacterial effects against bacteria (Branco et al., 2018; Pérez-Laguna et al., 2018; Tavares et al., 2018). PACT mediated by photosensitizers will produce ROS under specific wavelength light stimulation, which induces phototoxic damage to bacteria (Pérez-Laguna et al., 2019). PACT is not limited to its antibiotic resistance pattern to eliminate microorganisms and is also effective against microorganisms in a biofilm state (Giuliani, 2014; García et al., 2015).

Rose bengal (RB) is a xanthine dye used as a photosensitizer for PACT (Pérez-Laguna et al., 2017). The study found that RB-PACT combined with gentamicin has a synergistic antibacterial effect on planktonic *S. aureus*, while a synergistic effect is observed only when the maximum concentration tested of RB-PACT and gentamicin (64 μg/ml and 40 μg/ml, respectively) was used in biofilm (Pérez-Laguna et al., 2018). Its mechanism of synergistic antibacterial effect is considered to be that after PACT damages cells, it promotes the entry of gentamicin into bacteria, and gentamicin binds to the 30S subunit of the bacterial ribosome to impair protein synthesis, resulting in bacterial death (Barra et al., 2015; Pérez-Laguna et al., 2018).

The photobacterial agent toluidine blue (TB) is a hydrophilic cationic alkaline photosensitizer (PS). TB has high affinity for bacterial membranes and is believed to cause membrane damage (Wainwright et al., 2016). Further studies found that TB-PACT combined with gentamicin had a more significant synergistic antibacterial effect against *S. aureus* and MRSA than either treatment alone (Liu et al., 2020). Antimicrobial photodynamic therapy (aPDT) is considered to promote the passage of antibacterial drugs by destroying the outer membrane of bacterial cells using light irradiation. Blue light-emitting diode (LED) light irradiation affects the growth of both gram-negative and gram-positive bacteria.

When blue LED light was used in combination with the AGAs such as amikacin and gentamicin, it showed a synergistic antibacterial effect on *E. coli* and *S. aureus*, and promoted the inactivation of the bacteria, which might be attributed to the antibacterial effect induced by oxidative stress (Silva et al., 2019).

## 5 Conclusion

AGAs are widely used in clinical practice due to their advantages of good water solubility, broad antibacterial spectrum and strong antibacterial activities. With its wide applications, the appearance of side effects as well as the increasingly bacterial resistance has restricted their clinical application. In recent years, based on continuous research on AGAs providing a deeper understanding of the antibacterial mechanism of action and the resistance mechanism, the strategies to reverse bacterial resistance to AGAs is developing. The strategies include structural modification of older AGAs based on new target prediction of existing AGAs, hoping to restore their antibacterial activity and presenting the new charm of existing AGAs.

AGAs in combined with other antibacterial, anti-inflammatory, and analgesic drugs, natural plant extracts, and other ingredients have a synergistic antibacterial effect that allows reduction the dosage of single drugs and exert good antibacterial activity with lower side effects. Because many drugs with theoretical basis have synergistic antibacterial effect in the experimental stage, drug combinations are expected to improve the increasingly severe bacterial drug resistance, thereby ensuring a bright future for AGAs.

The combination of antibiotics and other drugs is an important strategy to overcome bacterial resistance. We hope that the prediction of the old AGAs drug targets and the investigation of the important mechanisms of bacterial growth will lay a foundation for finding new AGAs derivatives and compounds combined with AGAs.
